# The influence of bath and probe sonication on the physicochemical and microstructural properties of wheat starch

**DOI:** 10.1002/fsn3.1111

**Published:** 2019-06-13

**Authors:** Maryam Jamalabadi, Solmaz Saremnezhad, Akbar Bahrami, Seid Mahdi Jafari

**Affiliations:** ^1^ Graduated from the Center for Food and Hospitality Management, Department of Culinary Arts and Food Science Drexel University Philadelphia Pennsylvania; ^2^ Department of Food Science and Technology, Faculty of Advanced Sciences and Technology, Pharmaceutical Sciences Branch Azad University (IAUPS) Tehran Iran; ^3^ Program of Applied Science and Technology, The Center for Excellence in Post‐Harvest Technologies North Carolina A&T State University Kannapolis North Carolina; ^4^ Department of Food Materials and Process Design Engineering Gorgan University of Agricultural Sciences and Natural Resources Gorgan Iran

**Keywords:** microstructure, physicochemical properties, ultrasound, wheat starch

## Abstract

Ultrasound has been rapidly applied successfully in diverse food technological aspects including improvement of functional properties of food ingredients such as starch. This work was carried out to compare the influence of two types of sonication, bath and probe, on several physicochemical and microstructural properties of wheat starch. Two sonication probes (200 and 400 W) and a sonication bath (690 W) were applied to treat wheat starch suspensions at 15 and 30 min. Sonication time in 400 W probe was intermittent while for the other treatments, it was continuous. Swelling capacity (SC), solubility (SB), turbidity (TB), and oil absorption (OA) parameters were investigated for native (control) and sonicated wheat starch. Moreover, scanning electron microscopy (SEM) was performed to determine the influence of sonication treatments on the morphology of wheat starch granules. The highest level of SB and OA, as well as the lowest SC, was obtained for starch samples treated with 200 W sonication probe, while no significant difference (*p* < 0.05) was observed between two sonication times (15 and 30 min). The SEM images showed significant and nonuniform impact of ultrasound on the structure of starch granules in which some granules remained almost smooth, but some showed high irregular surfaces or even in some cases structure collapse. The highest disintegration of granules was obtained in probe 200 W treatments.

## 1 INTRODUCTION

Starch is a homo‐polysaccharide storage carbohydrate, widely distributed in plants, particularly cereals such as corn, potato, wheat, and rice. The amylose (amorphous region) and amylopectin (crystalline region) chains are the composing units of semicrystalline structure of starch (Ganje, Jafari, Tamadon, Niakosari, & Maghsoudlou, 2019). The ratio of amylose to amylopectin in most starches varies from 20% to 25% for amylose and from 75% to 80% for amylopectin, and this ratio is affected by the parameters such as botanical source, alterations among cultivars of every species, and the level of plant's maturity (Jambrak et al., 2010; Wu, Du, Ge, & Lv, 2011). Wheat is an important cereal that its starch content accounts for 75% of the grain weight (Shewry, 2009) and is greatly used for producing starch.

Starches due to their diverse techno‐functional properties are widely used in the industry for diverse applications, such as for improving viscosity, swelling and pasting properties, and digestibility. Although starch is available abundantly in quantity, inexpensive, environmentally degradable, free of hazardous contaminants, and renewable (Shabana et al., 2018), some drawbacks limits its commercial application such as insoluble in cold water, unstable in acid, resistant to dehydration, and having low emulsifying capacity. Therefore, starch modification is an important process, which is used to improve starch techno‐functional properties and broaden its applications in different industries including the food industry. In general, chemical, biological, and physical modifications are considered as three major starch modification techniques. Among physical methods, ultrasonic modification due to being eco‐friendly, highly efficient, and safe was the focus of many researches in recent years (Gaquere‐Parker et al., 2018; Li, Li, & Zhu, 2018; Shabana et al., 2018).

Ultrasound that attracted much attentions in the field of food processing is defined as the technology of using sound waves with a frequency higher than the typical human hearing range, that is, >15–20 kHz (Jafari, He, & Bhandari, 2006; Jalili, Jafari, Emam‐Djomeh, Malekjani, & Farzaneh, 2018). In order to use ultrasound in starch modification, either the native starch solution or starch after gelatinization can be subjected to the ultrasound (Zuo, Knoerzer, Mawson, Kentish, & Ashokkumar, 2009). The sonication of native starch changes the physicochemical (PH‐CH) characteristics of starch, in which its level is affected by several factors, such as the characteristics of prepared dispersion (e.g., botanical origin and concentration), ultrasound power and frequency, and time and temperature of modification (Zuo et al., 2009). Former studies explained two principal mechanisms associated with ultrasound technology. Cavitation is the first and more important mechanism resulting in the creation of gas bubbles (Jafari, He, & Bhandari, 2007) which bombards starch granules within the suspension medium before they collapse (Li et al., 2018). Moreover, rapidly collapsing bubbles releases a high energy which greatly increases pressure and temperature and results in the breaking of a fraction of bonds in starch structure and this breakdown imposes PH‐CH impacts. The second mechanism is explained by dissociating the water molecules to form free radicals (such as –OH and –H), that would induce starch polymer degradation and causes chemical alterations (Monroy, Rivero, & García, 2018; Shabana et al., 2018).

Several researchers have evaluated the effects of ultrasound on starches with various botanical origins such as corn, potato, sweet potato, and tapioca (Li et al., 2018; Shabana et al., 2018; Wang et al., 2017; Yang, Lu, Chen, Luo, & Xiao, 2018). The results of these studies have shown that sonication affected the PH‐CH, functional, and rheological characteristics of starch samples. For example, an increase in the SC was observed for the corn starch with the increase in ultrasound power and intensity (Wang, Cheung, Leung, & Wu, 2010). Manchun, Nunthanid, Limmatvapirat, and Sriamornsak (2012) reported that compared with native starch, the modified tapioca starch with ultrasound treatment came up with alterations in the molecular‐scale structure of starch, followed by significant variations in its physical properties. In particular, the SB as well SC of sonication‐treated starches increased (Manchun et al., 2012). Ultrasonication may also result in cracks and pores, and impose damages to the granules of starches with different botanical origins (Amini, Razavi, & Mortazavi, 2015; Falsafi, Maghsoudlou, Aalami, Jafari, & Raeisi, 2019). However, the type and structure of starch affects the level of its susceptibility to ultrasonication. For instance, for the same sonication conditions, the cracks and depressions on the granule surfaces of potato and wheat starches were deeper than rice and maize starches (Sujka & Jamroz, 2013). Furthermore, changes in the treatment parameters and overall conditions of sonication treatment (such as frequency, power, and treatment time) have different effect on the microstructure and PH‐CH properties of a specific starch. For example, dual‐frequency ultrasonication (20 and 25 kHz) has created more depression on corn granules than a single frequency ultrasound (Hu et al., 2015).

The effects of sonication on modification of starch through different ultrasound devices (bath and probe sonication) and continuous sonication compared with intermittent sonication has rarely been studied. Therefore, this work was aiming to examine the influence of different sonication devices (probe and bath) with different powers and different treatment times (continuous vs. cycle set) on PH‐CH and microstructure properties of wheat starch.

## 2 MATERIALS AND METHODS

### 2.1 Preparation of starch suspensions

Powdered wheat starch (Analytical grade starch, 21,000, purity >98%, pH: 7, water: 11%; MERK Co) was used in all experiments. The suspensions were produced by suspending 10 g wheat starch in 90 ml distilled water to provide 10% (W/W) samples. Then, the suspensions were mixed on a magnetic stirrer (IKA RCTBASIC) at speed 1,500 rpm for 15 min and utilized in the following steps.

### 2.2 Ultrasound treatments of wheat starch

For sonication treatment of wheat starch suspensions, firstly, two types of ultrasound probe devices with 24 kHz frequency (UP 200S and UP 400S) were applied. 500 ml volume of prepared samples was placed in a conical flask. Ultrasound probe equipped with a vibrating titanium tip (diameter: 8 mm) was immersed into the suspension container and each sample was sonicated continuously (200 W) or intermittently (400 W) for 15 and 30 min, while the amplitude was set at 100%. In other words, for the two 400 W samples, the intermittent condition, instead of continuous sonication was applied. For this, after every 5 min of treatment, one minute resting time was considered. For bath sonication, 500 ml of starch samples were poured directly into the ultrasound bath (Sharp, UT 606H) and treated with ultrasound waves (40 kHz frequency and 690 W power) for 15 and 30 min. Different treatments and their relevant conditions have been depicted in Table 1.

**Table 1:** Different treatments and their conditions for sonication of wheat starch samples

Sample code	Ultrasound device	Power (W)	Time (min)	Sonication conditions
C	Untreated (control)	—	—	—
P_200,15_	Probe	200	15	Continuous
P_200,30_	Probe	200	30	Continuous
P_400,15_	Probe	400	15	Intermittent
P_400,30_	Probe	400	30	Intermittent
B_15_	Bath	690	15	Continuous
B_30_	Bath	690	30	Continuous

### 2.3 Freeze‐drying of sonicated starch suspensions

Upon the end of ultrasound treatment, the starch samples were dried by using a laboratory scale freeze dryer (Christ‐Alpha 1–2 kg –LD). The dishes containing the starch suspensions (0.12 m^2^ × 12 cm) were placed on the trays of the freeze dryer (−55°C) for 6 hr (<100 Pa) to freeze and dry the starch samples.

### 2.4 Evaluation of physicochemical characteristics of sonicated starches

#### 2.4.1 Swelling capacity and solubility

The Swelling capacity (SC) and solubility (SB) of the starch samples were analyzed according to the method described by Chang, Lin, and Chang (2006). Firstly, the starch samples were weighed (*W*_0_) inside a tube and 10 ml distilled water was added. Then, the tubes were heated in a water bath (WNB, Memmert) at 85°C for 25 min. The tubes were cooled to room temperature and centrifuged (Heraus Christ‐Multifuge x1) at 2,200 rpm for 20 min. The supernatant samples were dried to constant weight (*W*_1_) using a vacuum oven (Fischer Scientific) at 110°C for 5 hr. The wet starch sediments were also weighed (*W*_2_) to measure the SC of starch samples. The experiments were done in triplicates. The SB and SC were determined using the following equations (Jambrak et al., 2010):

(1) $$\text{SB}\left(\%\right)=\frac{W_{1}}{W_{0}}\times 100$$

(2) $$\text{SC}\left(\%\right)=\frac{W_{2}}{W_{0}}\times 100=\left(100-\text{SB}\right)$$

#### 2.4.2 Turbidity

The turbidity (TB) of sonicated starch samples was measured according to the method suggested by Jambrak et al. (2010). Firstly, a 1% (w/v) water suspension of wheat starch was heated at 85°C near neutral pH using a water bath for 1.2 hr with constant stirring. Then, after cooling the suspensions to 32°C, the TB was obtained by reading the absorbance at 650 nm against water blank in 1 cm path length cuvette, using a Ultraviolet–visible spectrophotometer (Reyleigh‐UV 1800). Finally, the TB was obtained using the Equation 3:

(3) TB=2303∗A/I

TB, turbidity; *A*, the absorbance at 650 nm; *I*, the path length of cuvette (m).

#### 2.4.3 Oil absorption

For this index, 10 ml rapeseed oil was added to the tubes containing weighted starch powder samples (1 g) and the tubes were homogenized at 1,000 rpm for 1.5 min (Heraus Christ‐Multifuge). After 5 min, the samples again were homogenized at 1,700 rpm for 12 min. Then, the unabsorbed oil was rinsed and the weight of absorbed oil was determined (Sujka & Jamroz, 2013).

### 2.5 Evaluation of the microstructure of sonicated starch samples

The samples were dehydrated in 99.5% ethanol and scattered on double‐sided tape and, then, were seated on an aluminum stub. Subsequently, the coating was performed with a thin gold film using sputter coater and observed in a scanning electron microscope (SEM; model KYKY‐32000), under accelerating potential of 30 kV.

### 2.6 Statistical analysis

All treatments were triplicated, and values were representative of the mean of three measurements. The impact of different sonication treatments on starch properties was investigated with the ANOVA (analysis of variance), using SPSS version 21 (IBM). The significant differences among means was determined by Duncan's test, performed at *α* = 0.05 significant level.

## 3 RESULTS AND DISCUSSION

### 3.1 Swelling capacity and solubility of sonicated starches

The SC and SB are two key factors which provide information on the quantity of interactions between starch chains associated with the amorphous and crystalline regions. The degree of this interaction is affected by the numerous parameters such as the ratio of amylose/amylopectin and the branching level and length of the amylose and amylopectin (Singh & Kaur, 2004). The SC of a starch sample is defined as its molecule's capacity to keep water in the starch structure with hydrogen bonds (Bashir, Jan, & Aggarwal, 2017). The rheological properties of starch, such as its thickening behavior, are greatly affected by the degree of SC and SB of starch (Desam, Li, Chen, Campanella, & Narsimhan, 2018; Tafti, Peighambardoust, Behnam, et al., 2013; Tafti, Peighambardoust, Hesari, Bahrami, & Bonab, 2013). For example, reduced amount of granule SC and SB due to modification process applied was associated with the decrease in the paste viscosity and clarity, and the amylose leaching level of cross‐linked maize starch (Desam et al., 2018).

**Figure 1:**
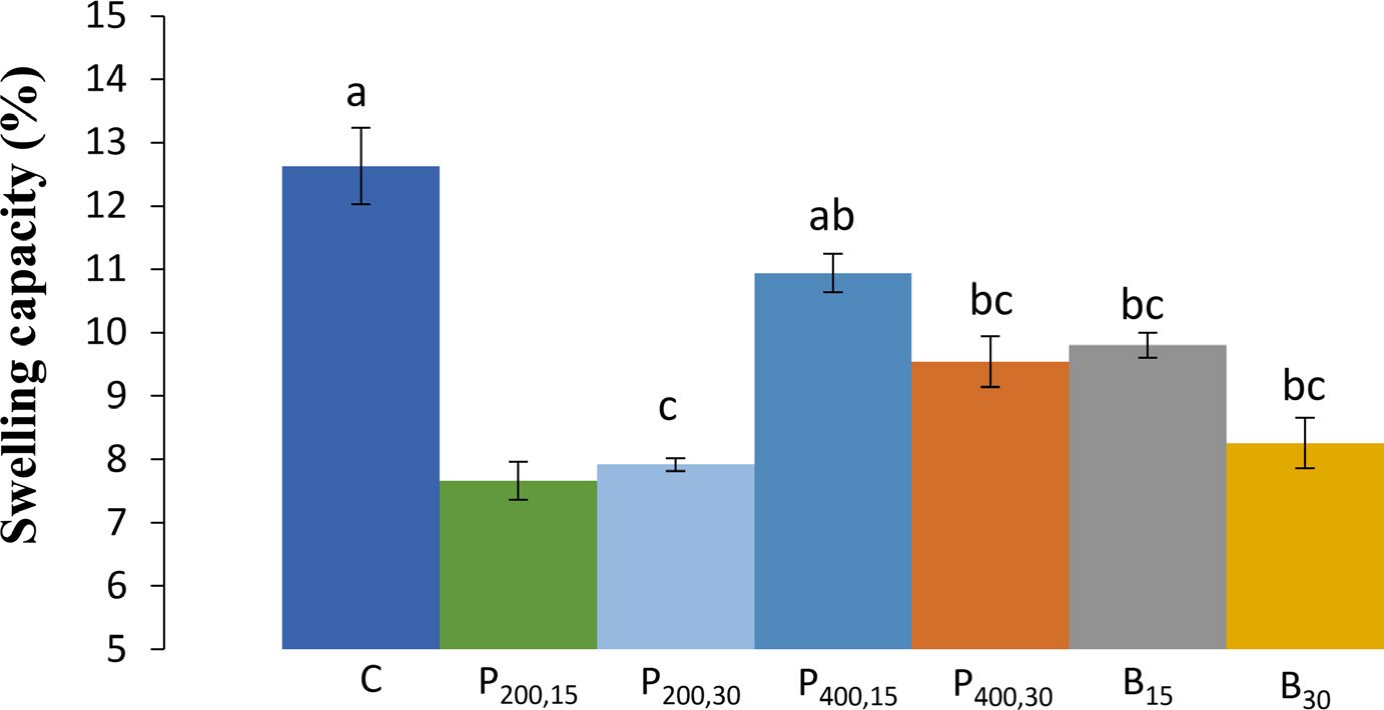
Values of the swelling capacity (%) for the wheat starch‐sonicated samples. C (control, untreated sample); P_200,15 _(ultrasound probe—200 W–15 min); P_200,30_ (ultrasound probe—200 W–30 min); P_400,15_ (ultrasound probe—400 W–15 min); P_400,30_ (ultrasound probe—400 W–30 min), B_15_ (ultrasound bath—690 W–15 min); B_30_ (ultrasound bath—690 W–30 min). Different letters (a, b, c) indicate a significant difference (*p* < 0.05) among samples

The SC of untreated and sonicated wheat starches are shown in Figure 1. Ultrasound treatment significantly decreased (*p* < 0.05) the SC for all samples, compared with the control sample (with a SC of 12.63%), except for the sonication probe 400 W for 15 min, which the decrease in its SC was not statistically significant (*p < *0.05). The highest reduction in the SC was obtained for the sonication probe 200 W samples (7.66% and 7.91%, for 15 and 30 min, respectively), followed by the sonication bath treatment for 30 min (8.25%). The lower effect of ultrasound treatment at higher intensity (400 W), compared with the lower intensity (200 W) on the SC could be due to the intermittent conditions used in 400 W probe sonication, compared with the continuous application of probe sonication for the 200 W samples. The continuous sonication resulted in a higher temperature, rather than the intermittent use, even in higher intensities (400 W).

The decrease in the SC of sonicated starches might be due to the structural fragmentation of starch granules during the ultrasound process (as observed in SEM images) and loosening of starch polymer network (Falsafi et al., 2019), resulted in the negative impacts on the SC. Furthermore, it was found that in the samples with a lower SC, there was a higher increase in temperature compared with control sample. Therefore, it can be concluded that the increase in temperature (reaching to the 65–95°C) contributed to the decrease in SC directly or indirectly by facilitating the granule structure degradation (Bashir et al., 2017). Our results were inconsistent with the reports on the sago starches subjected to the ultrasound, and a significant decrease in their SC compared with the native starches was found (Chan, Bhat, & Karim, 2010). However, some other researchers have reported a raise in the SC for ultrasound‐treated starches (Monroy et al., 2018; Sujka & Jamroz, 2013) which these results were mainly attributed to the facilitation of the water penetration into the granules due to starch gelatinization and weakening of hydrogen bonds.

**Figure 2:**
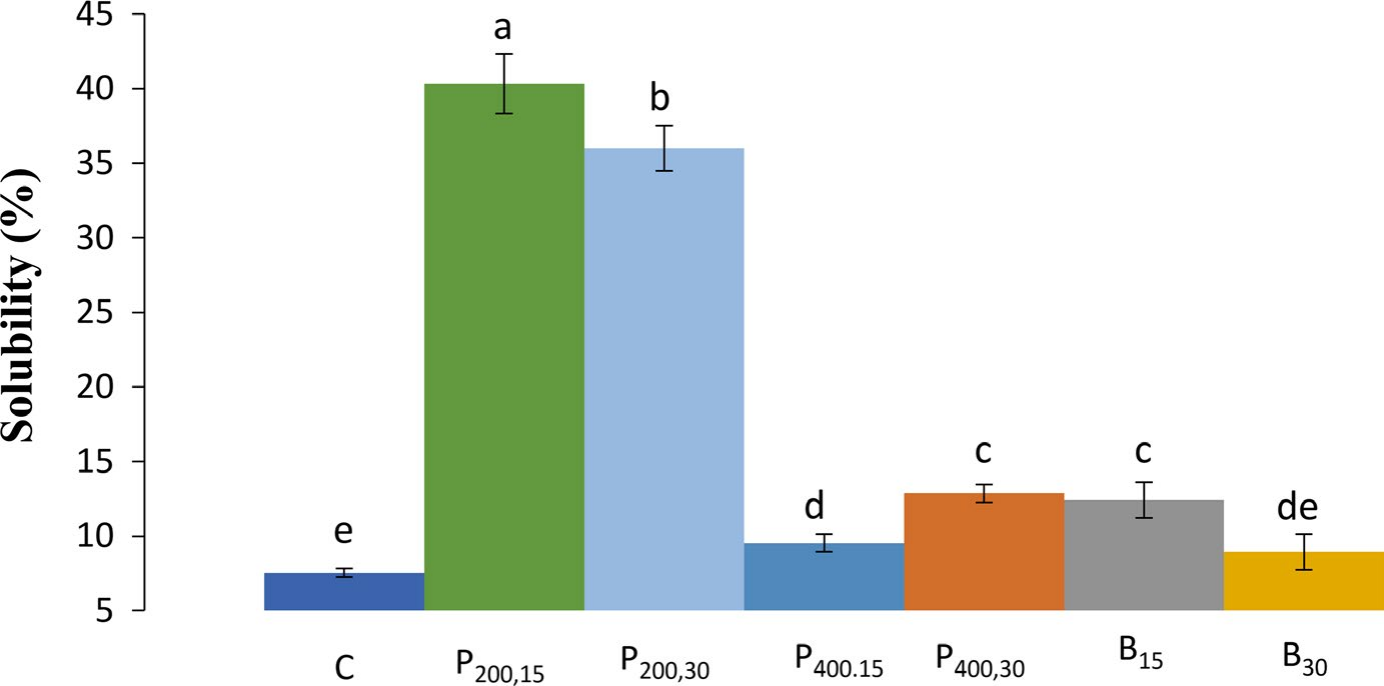
Values of the solubility (%) for the wheat starch‐sonicated samples. C (control, untreated sample); P_200,15 _(ultrasound probe—200 W–15 min); P_200,30_ (ultrasound probe—200 W–30 min); P_400,15_ (ultrasound probe—400 W–15 min); P_400,30_ (ultrasound probe—400 W–30 min), B_15_ (ultrasound bath—690 W–15 min); B_30_ (ultrasound bath—690 W–30 min). Different letters (a, b, c) indicate a significant difference (*p* < 0.05) among samples

According to Figure 2, ultrasound significantly (*p* < 0.05) raised the SB of wheat starch samples, compared with the control sample (7.53%), except for the ultrasound bath for 30 min, which its influence was not statistically significant (8.94%). The two highest SB values were obtained for the ultrasound probe 200 W samples (40.32 and 35.99% for 15 and 30 min, respectively). Similar to the SC results, the lower effect of ultrasound at higher intensities (400 W) compared with the lower intensities (200 W) might was due to the intermittently use of probe sonication at 400 W and a The major changes on the starch granules caused by ultrasound are through the cavitation forces, which break down the crystalline molecular structure and the chains of starch by disrupting bonds (Huang, Li, & Fu, 2007; Jambrak, Mason, Lelas, Herceg, & Herceg, 2008). These changes would increase the possibility of water to enter into the structure of granules and bind to the free hydroxyl groups, resulted in an increase in the SB of starch. Similarly, a progressive raise in the SB for all of potato, corn, bean, sago, mung (Chan et al., 2010), and oat starches (Falsafi et al., 2019) after sonication treatment was observed. That was demonstrated that the depolymerization and weakening of the structure of starch due to ultrasound was the main cause of the increase in the SB (Chan et al., 2010; Sujka & Jamroz, 2013). Besides the degradation of granular structure, the formation of linear fractions (with low molecular weight) due to the release of side chains of amylopectin or cleavage of chains of amylose was the cause of increase in the SB level of starch samples subjected to ultrasound (Amini et al., 2015; Falsafi et al., 2019). Similarly, Zhu (2015) concluded that the ultrasound raised the SB of starch samples, but, had positive, negative, or inert impact on the SC of starches. Also that was demonstrated that the ultrasound impact was affected by diverse parameters such as, frequency, intensity, time, temperature, and the water level of the starch solution (Zhu, 2015).

### 3.2 Microstructure of sonicated starch samples

**Figure 3:**
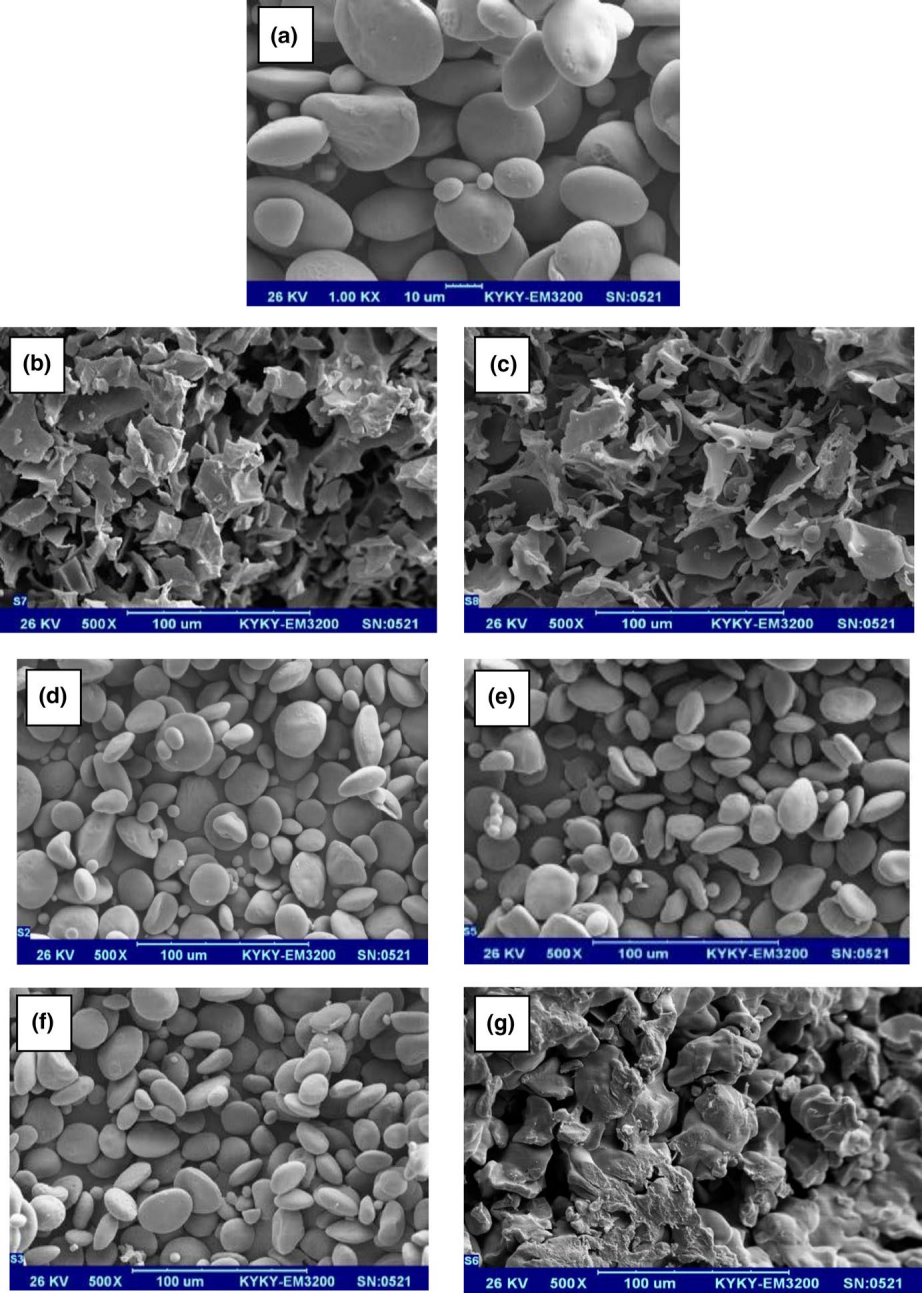
SEM images for the wheat starch‐sonicated samples. (a) control, untreated sample; (b) ultrasound probe, 200 W–15 min; (c) ultrasound probe, 200 W–30 min; (d) ultrasound probe, 400 W–15 min; (e) ultrasound probe, 400 W–30 min; (f) ultrasound bath, 690 W–15 min; (g) ultrasound bath, 690 W–30 min

The SEM images provide the opportunity to study the morphological alterations on target starch granules subjected to different processes. Pictures from micrographs of wheat starch and sonicated samples are shown in Figure 3. In these images, the significant impact of ultrasound on the structure of granules is clear. Besides, ultrasound reduced the size of wheat starch granules, compared with the control treatment which was in agreement with the reports of Falsafi et al. (2019). As can be seen in Figure 3a, the granules of native wheat starch had a smooth surface without pores and fissure. Native starch granules were in an aggregated structure in the shape of clusters, and individual granules were mostly irregular in shape, but ovoid‐like in some cases. The SEM images of ultrasound treatments revealed that the granules of different samples were not affected uniformly by various sonication treatments, even in every sample, granules were affected differently. While some granules remained almost smooth after sonication, some showed high irregular surfaces or even in some cases their structure has been collapsed. Therefore, the great level of structural damages was an indicator of the alterations in the physicochemical, as well as the functional properties of wheat starch samples subjected to ultrasound.

The SEM analysis indicated that the surface of granules subjected to the ultrasound probe 400 W exhibited only fine fissures (Figure 3d,e), while the samples sonicated by probe 200 W (Figure 3b,c) showed a high degree of decomposition of granular structures. The reason for this difference probably was the continuous application of ultrasound probe for samples at 200 W intensity, compared with intermittent (cycles) sonication the 400 W intensity. The continuous sonication resulted in a higher temperature, thus, more gelatinization and the profound collapse of granules occurred. The SEM images also showed that the starch samples subjected to ultrasound bath for 15 min presented some irregularities in the surface of some granules and also some particles had a depth‐concave geometry (Figure 3f). However, the decomposition of granular structure for ultrasound bath at 30‐min sonication (Figure 3g) was found to be similar to 200 W probe sonication samples. Thus, the changes in the duration of bath ultrasound greatly affected the structure of wheat starch granules.

The impacts of ultrasound on starch granular morphology were attributed to the appearance and destruction of bubbles (Hu et al., 2015). The bubble's collapse induces a high‐pressure gradient in high local velocities which makes shear forces that are the main cause of fracturing the polymer chains and granules damages (Hu et al., 2015; Yang et al., 2018). The other associated phenomenon is that the ultrasound partially decomposes water into hydroxyl and hydrogen due to the collapse of produced bubbles. These free radicals by coming out of the cavities and entering into the surrounding can make some variations in the PH‐CH characteristics of starch (Yang et al., 2018). These results were in agreement with the studies of Hu et al. (2015) and Yang et al. (2018) who found noticeable pores and fissures on the surface of starch granules due to the impacts of ultrasound. The significant defects on the potato granular structures were reported by Zuo, Hebraud, Hemar, and Ashokkumar (2012). In a similar work (Jambrak et al., 2010), it was concluded that the bath and probe sonication provided different outcomes for a specific starch, and the higher power input raised the possibility of granules agglomeration.

### 3.3 Turbidity of sonicated starch samples

The TB of starch pastes is considered as an important characteristic regarding their application in food processes, especially for some types of products such as fruit fillings or clear fruit juices in which a lower TB is more acceptable. In such these cases, a high level of TB is an unwilling property; thus, commonly several modified or waxy starches are used to reduce or prevent the TB. The extent of amylose and its smaller chains (a feature that eases alignment of linear chains) are important parameters affecting the starch paste TB. Researchers reported that separation of several heterogeneous materials (such as sugars and salts) during starch modification through physical technologies might be the main cause of alterations in color and clarity levels of starches which are closely associated with the TB (Falade & Ayetigbo, 2015).

**Figure 4:**
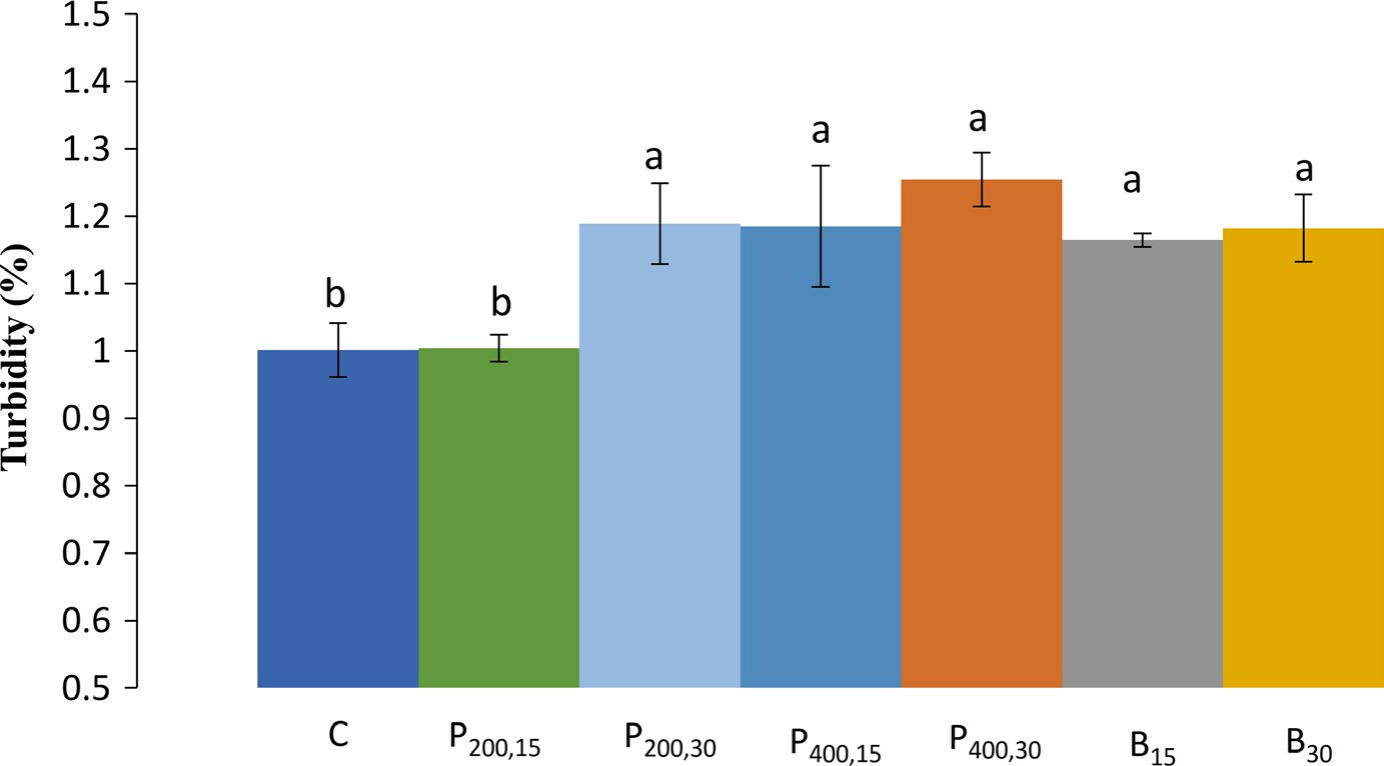
Values of the turbidity (%) for the wheat starch‐sonicated samples. C (control, untreated sample); P_200,15 _(ultrasound probe—200 W–15 min); P_200,30_ (ultrasound probe—200 W–30 min); P_400,15_ (ultrasound probe—400 W–15 min); P_400,30_ (ultrasound probe—400 W–30 min), B_15_ (ultrasound bath—690 W–15 min); B_30_ (ultrasound bath—690 W–30 min). Different letters (a, b, c) indicate a significant difference (*p* < 0.05) among samples

The results of TB experiment for the control and sonicated starches presented a significant (*p* < 0.05) effect of ultrasound on the wheat starch TB, as shown in Figure 4. Samples from all sonicated starches showed a significantly (*p* < 0.05) higher TB than the native starch, except for the treatment of ultrasound probe 200 W for 15 min, which its TB was similar to the native starch (1.00%). The sample sonicated with probe 400 W for 30 min presented the highest TB (1.25%). Similarly, Sujka and Jamroz (2013) observed that sonication in ethanol caused a small increase in the paste TB for wheat and rice starches, but no significant changes for the corn and potato starches were observed.

The decrease in paste clarity could be due to the rearrangement of starch granules which can induce a higher level of light absorption (Zuo et al., 2012). Furthermore, leaching of amylose and amylopectin chains resulting in higher levels of light absorbance can also be an important factor affecting the TB (Jan, Panesar, Rana, & Singh, 2017). However, some researchers have shown that the clarity for the sonicated starches increased, because the swollen granules were disrupted in a great extent during the process (Hu et al., 2015; Jambrak et al., 2010).

### 3.4 Oil absorption of sonicated starch sample

**Figure 5:**
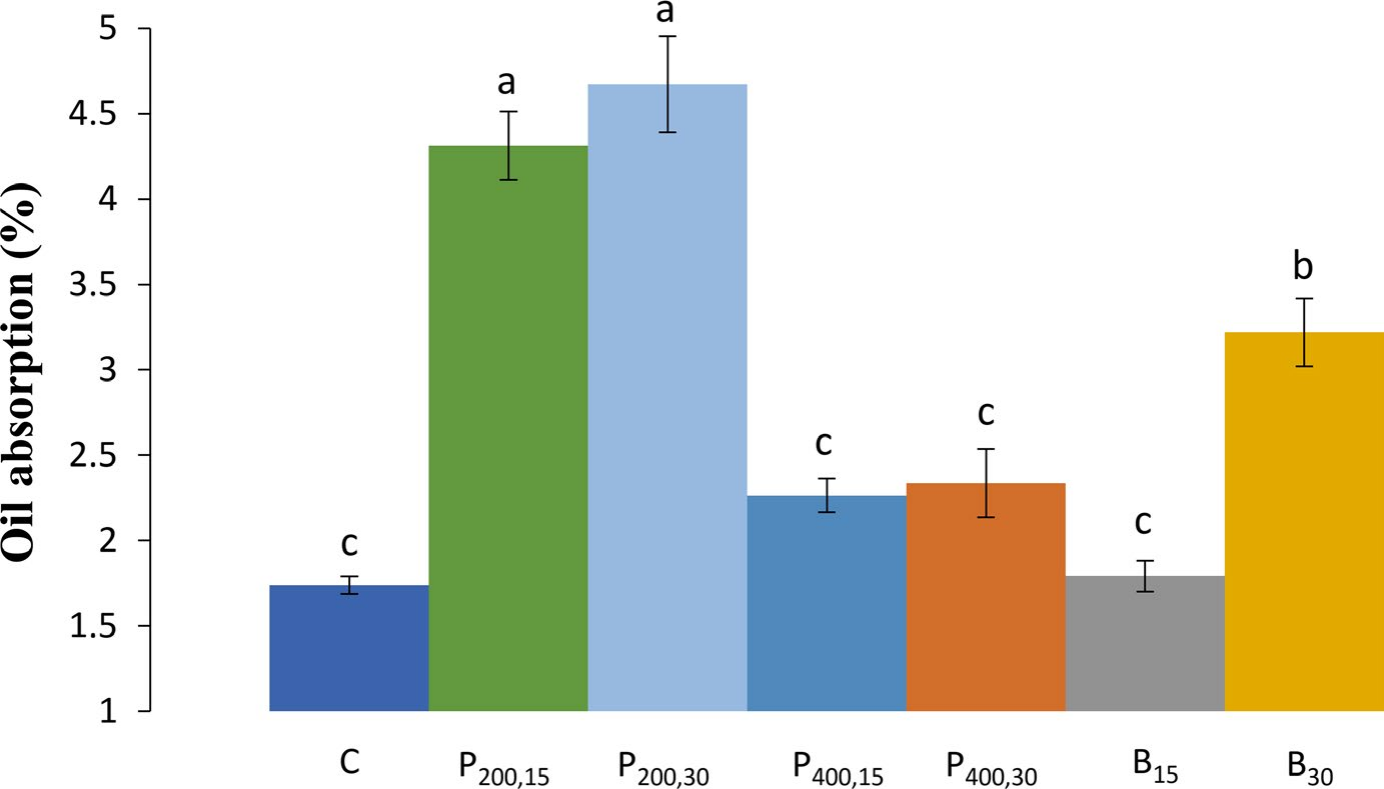
Values of oil absorption (%) for the wheat starch‐sonicated samples. C (control, untreated sample); P_200,15 _(ultrasound probe—200 W–15 min); P_200,30_ (ultrasound probe—200 W–30 min); P_400,15_ (ultrasound probe—400 W–15 min); P_400,30_ (ultrasound probe—400 W–30 min), B_15_ (ultrasound bath—690 W–15 min); B_30_ (ultrasound bath—690 W–30 min). Different letters (a, b, c) indicate a significant difference (*p* < 0.05) among samples

The oil absorption (OA) is a physical entrapment which is dependent on the size as well as the shape of starch granules (Verma et al., 2018). The ultrasound significantly (*p* < 0.05) increased the OA for the wheat starch samples sonicated with the probe 200 W for 15 min and 30 min (4.31% and 4.67%, respectively), and the ultrasound bath for 30 min (3.22%), compared with the control sample (1.74%), as presented in the Figure 5. The increasing of OA for the other samples, the probe 400 W for 15 and 30 min (2.26% and 2.34%, respectively), and the ultrasound bath for 15 min (1.79%) did not show a statistically significant (*p* < 0.05) difference with the control sample. In a study by Sujka and Jamroz (2013), the ultrasound treatment increased the OA of the wheat, potato, corn, and rice starch granules in water and ethanol than those obtained for the native starches. In particular, the OA of wheat starch raised over 60% due to sonication (Sujka & Jamroz, 2013). Huang et al. (2007) showed that the OA was significantly improved with using ultrasound in combination with glucoamylase. They explained that sonication extended the surface as well as internal area of starch granules (e.g., pores and channels), in which this might was the reason of facilitated OA through the ultrasound modification. Furthermore, some researchers demonstrated that the cavitation and thermal effects of the ultrasound may result in the cutting out of the linear chains and decreasing the branch chains on the surface of starch granules, thus, increased the OA (Wu et al., 2011).

## 4 CONCLUSION

Ultrasound, compared with the traditional technologies used for starch modification, is much more energy efficient which increases the temperature of treatment medium in a lower extent. Our results showed an increase in the SB as well as the OA degree of sonicated wheat starch samples. The disruption of the granular structure resulted in a higher water and oil uptake might was the key cause of increased SB and OA levels due to sonication. The decrease in the SC for sonicated samples might was due to the increase in temperature and collapse of granules and led granules to lose their ability to swell. The SEM images showed the great mechanical damages imposed on starch granules in some of ultrasound treatments. This study by presenting thorough information on the changes in the structure and PH‐CH characteristics of wheat starch subjected to the different ultrasound treatment can be used as a base for further works. As the PH‐CH features of wheat starch profoundly varied due to the changes in ultrasound treatment conditions, more study needs to be carried out to optimize the process parameters and propose the best ultrasound conditions according to the initial starch properties. Ultrasound conditions, such as power and frequency, time, and temperature, are among important factors that should be optimized to inhibit or decrease adverse impacts of sonication.

## CONFLICT OF INTEREST

All authors declare that there is no conflict of interest.

## ETHICAL STATEMENT

There was no human or animal testing in this study.
